# The effect of staff training on welfare outcomes of free-roaming dogs in a catch-neuter-vaccinate-release facility in India

**DOI:** 10.1017/awf.2025.10049

**Published:** 2025-11-13

**Authors:** Aswin Susheelan, Emma Rayner, Luke Gamble, Marie Haskell

**Affiliations:** 1 Mission Rabies, Worldwide Veterinary Service, Mumbai, India, 400703; 2 Worldwide Veterinary Service, UK; 3 Animal and Veterinary Sciences, SRUC (Scotland’s Rural College), UK

**Keywords:** Animal welfare, assessment, CNVR, free-roaming dogs, score, staff training

## Abstract

Catch-neuter-vaccinate-release (CNVR) programmes for free-roaming dogs (FRD) are humane and effective in controlling dog populations in developing countries. However, each component, from capture to release, can impact an individual animal’s welfare. This study aimed to develop a standardised welfare assessment scoring system for evaluating the welfare of dogs undergoing routine CNVR procedures at a veterinary training facility in Goa, India and to assess the impact of a targeted staff training intervention by comparing welfare assessment scores before and after its implementation. A score-based protocol was designed, incorporating 22 animal-, resource-, and management-based parameters covering six key steps of the CNVR procedure: catching/transport; cage/holding area; pre-operative period; surgery; post-operative period; and release. Eighty-two dogs were assessed initially. Areas for improvement were identified and informed the design of a targeted staff training intervention involving theory-based lectures and interactive sessions. Knowledge was assessed before and after receiving the intervention, with total scores on the assessment improving. The welfare assessment was repeated on another 81 dogs. Total welfare scores for individual dogs improved significantly after the staff intervention compared with before. This study demonstrates that a welfare assessment tool can be used to assess the welfare of individual dogs in a busy CNVR programme; furthermore, it can inform the compilation and delivery of a targeted staff training intervention and demonstrate improvements in dog welfare after such an intervention.

## Introduction

The global population of free-roaming dogs (FRDs) is estimated to be between 500–700 million (Hiby & Hiby 2017). India is one of the countries with the highest number of FRDs with approximately 17.1 million dogs according to the 2012 livestock census (Lynteris 2019) and approximately 137,000 in Goa in 2017 (Gibson *et al.* 2022). Overall, a high FRD population can substantially compromise the welfare of individual animals, with problems reported that arise from poor health, high mortality, and human abuse (Bacon *et al.* 2017; Berteselli *et al.* 2021). Furthermore, the presence of FRDs is associated with the transmission of zoonotic diseases such as rabies, Leishmaniasis, echinococcus, and blood-borne parasites, and poses a threat to people as well as livestock and wildlife (Jackman & Rowan 2007; Hiby & Hiby 2017). Worldwide, rabies is the cause of approximately 55,000 human deaths per annum, most of them transmitted via dog bites (Gibson *et al.* 2015), with 20,000 of these occurring in India (Sudarshan 2006). Therefore, effective dog population management (DPM) strategies are critical in addressing these challenges. Implementation of such strategies has demonstrable benefits to both dogs and people. For individual animals, improvements in body condition scores (BCS), and a decrease in the prevalence of infectious diseases amongst the dog population, have been described (Hiby *et al.* 2017); and from a ‘one health’ perspective, there has been a reduction in both dog bite incidents and concomitant human deaths from rabies (Reece 2005; Byrnes *et al.* 2017).

Currently, surgical sterilisation remains the main control method promoted to manage FRD populations humanely and effectively (also referred to as trap-neuter-return [TNR], catch-neuter-vaccinate-return [CNVR] or animal birth control [ABC] programmes). CNVR programmes are often implemented by local government and animal welfare charities. They involve humane capture, surgical neutering under general anaesthesia, administration of a rabies vaccination and, after an appropriate post-operative recovery period, release back to their original location from where they were caught (Bacon *et al.* 2017). The global nature of the CNVR programmes, their application by animal welfare charities and governments, and the comparatively few welfare implications, all contribute to the public perception that CNVR is a welfare-friendly method of dog population control (Jackman & Rowan 2007; Bacon *et al.* 2017).

Until recently, the welfare experience of individual dogs undergoing the CNVR process has been largely overlooked, with success measured primarily by fixed targets, such as number of dogs neutered in a day (Jackman & Rowan 2007; Arena *et al.* 2018). A recent review highlighted key areas in the CNVR process that can compromise welfare (Bacon *et al.* 2017). Protocols have been developed for assessing the welfare of individual dogs undergoing CNVR specifically (Bacon *et al.* 2019a; Berteselli *et al.* 2021). To ensure high standards of welfare are maintained throughout the entire process, evaluation of each facility’s standard operating procedures is crucial. However, in addition, assessment of the staff is also crucial which, through the delivery of effective staff training, can affect the implementation of improved, humane catching and transport techniques, proper surgical procedures, and effective anaesthesia protocols with multimodal anaesthesia (Bacon *et al.* 2017). This highlights the need for investment in staff training and education in CNVR facilities, informed by such evidence-driven methods.

The aim of this study was to develop a welfare assessment protocol for dogs undergoing the CNVR process from capture through to release in a CNVR training centre in Goa, India and to evaluate the impact of a staff training intervention on the welfare experience of individual dogs. A comprehensive welfare assessment was compiled and conducted on a sample of dogs undergoing CNVR at a spay-neuter training centre in Goa, India.

## Materials and methods

### Ethical approval

This study was approved by the University of Edinburgh Royal (Dick) School of Veterinary Studies’ Veterinary Ethical Review Committee (VERC 88.21). Ethics approval was not sought from the Human Research Ethics Committee, as the training constituted part of the training centre’s routine staff development activities. Furthermore, all data pertaining to staff training were anonymous and personal details were not collected at any point. All participants were informed about the purpose and scope of the study prior to the start of the training. Oral consent was obtained from each participant, acknowledging their awareness and agreement to participate in the study. Written consent was not deemed necessary, as the training formed part of the organisation’s ongoing professional development programme and is a standard requirement for staff involved in CNVR procedures.

### Study location: Overview

The location for this study was the Worldwide Veterinary Service’s (WVS) International Training Centre in Goa, India (ITC Goa) between October 2021 and January 2022. The centre conducts regular, two-week training courses providing veterinary participants with practical experience undertaking dog spay neuter surgeries on FRDs. These are carried out under the direct supervision of WVS staff veterinarians according to the latest spay-neuter guidelines and international standards (Airikkala-Otter *et al.* 2018; Rayner *et al.* 2019), thereby advocating high welfare standards throughout the process from capture to release. In addition to the training, WVS staff veterinarians also routinely perform sterilisation surgeries on free-roaming dogs and cats, averaging around 300 dogs and 100 cats per month.

### Study design

A comprehensive welfare assessment tool was developed to evaluate the physical and behavioural welfare of dogs at each of the six key steps of the CNVR process: catching and transport; cage/holding area; pre-operative period; surgery; post-operative period; and release. In the initial phase, 82 dogs undergoing routine CNVR process were assessed using this scoring system to establish baseline welfare standards. Parameters that contributed to reduced welfare were identified and categorised according to the stage of the process in which they occurred.

Based on the findings from this initial assessment, a targeted training intervention was designed and delivered to the ITC staff. The training aimed to address the specific areas where welfare compromises had been observed, with a focus on improving handling techniques, pre- and post-operative care, and overall awareness of animal welfare best practices. Following the training intervention, a second sample of 81 dogs was assessed using the same welfare scoring system. The welfare scores from this post-training group were then compared to the baseline scores recorded prior to the intervention to evaluate the effectiveness of the staff training on improving animal welfare outcomes.

**Figure 1.**
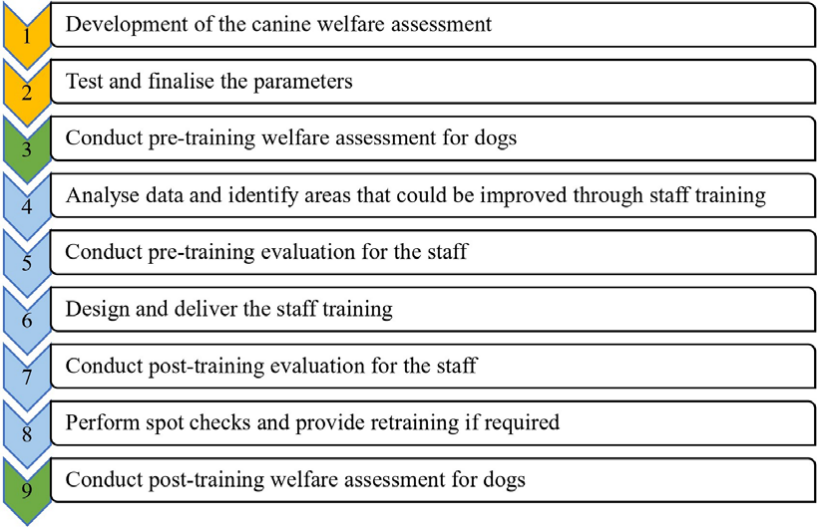
Flow-chart summarising the steps in a catch-neuter-vaccinate-release (CNVR) procedure in a study evaluating the impact of staff training on welfare outcomes of free-roaming dogs in a training facility in India. Blue denotes staff-training intervention, green denotes canine welfare assessment and orange denotes development/testing phases of the welfare protocol.

The study methodology is outlined in Figure 1 and each stage described below.

### Development and testing of the dog welfare assessment (stages 1 and 2)

**Figure 2.**
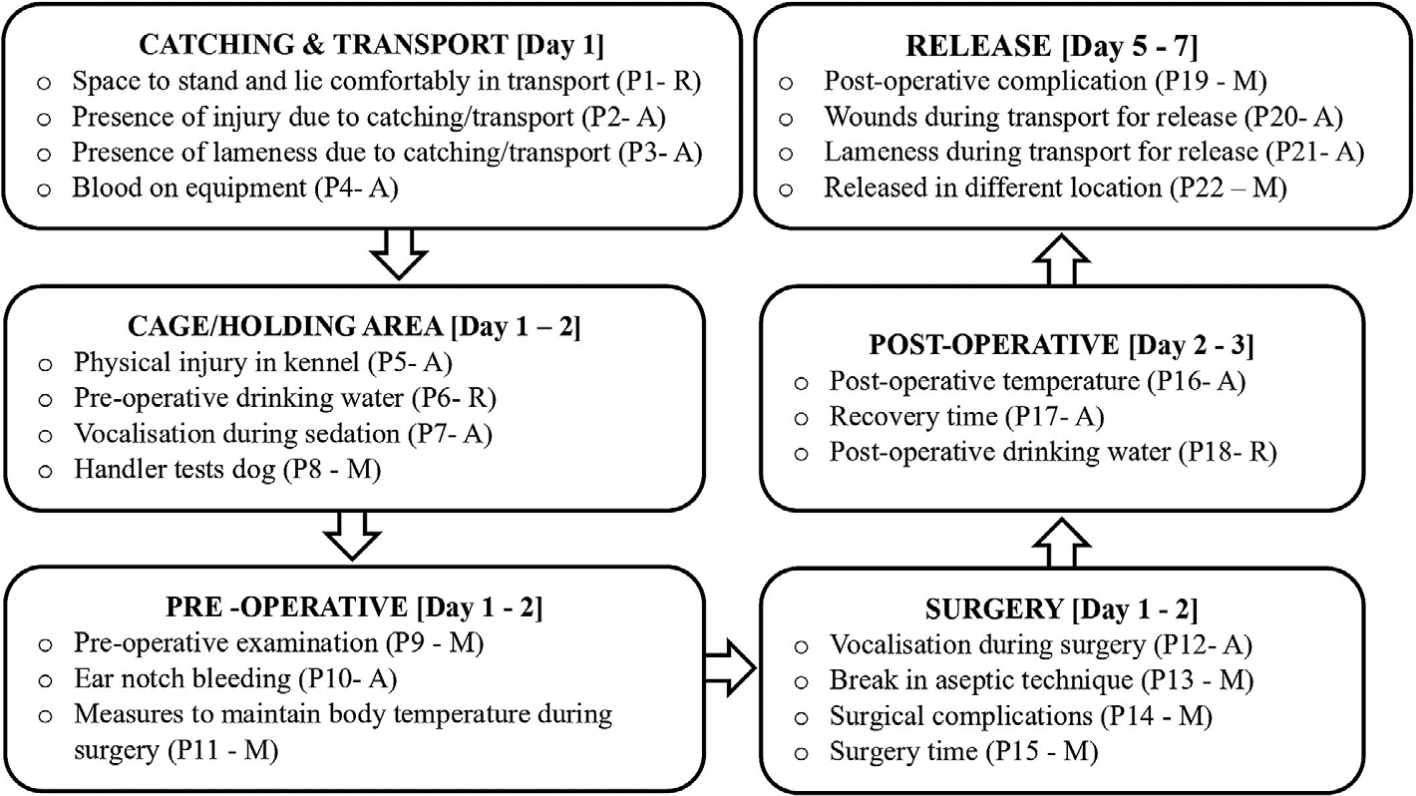
Flowchart showing the welfare assessment parameters associated with each step in a CNVR procedure in a study evaluating the impact of staff training on welfare outcomes of free-roaming dogs in a training facility in India. The parameter number is shown (e.g. P1, P2) followed by the sub-classification: A = Animal-based; R = Resource-based; M = Management-based.

The first stage of the study was to design a welfare assessment protocol specific to the facility. Animals exposed to stressors, such as those present within a shelter environment (Arena *et al.* 2018; Berteselli *et al.* 2019), seek to return to homeostasis through physiological, psychological, and behavioural responses (Barnard *et al.* 2016). These responses can be assessed using animal- (ABM), resource- (RBM), and management-based measures (MBM) (Barnard *et al.* 2014). A total of 22 such parameters that were considered relevant for the assessment in the specific setting of ITC Goa, were identified (Figure 1; stage 1 and Figure 2). MBM and RBM investigated the policies, resources, programmes, and practices of the institution (Kagan *et al.* 2015), while ABMs indicated the outcome from the inputs provided (Velarde & Dalmau 2012). Sixteen parameters were selected from Bacon *et al.*’s (2019a) study, and the remainder from the following sources: a range of published literature (Barnard *et al.* 2016; Airikkala-Otter *et al.* 2018; Berteselli *et al.* 2021), the first author (AS’s) experience working with CNVR programmes, and discussion with colleagues employed in this field (Appendix 1; Supplementary material). The parameters evaluated the six key steps in the CNVR process (Figure 2). A scoring system was then developed that converted each of the selected welfare measures to meaningful welfare scores. To maximise standardisation, the scoring of the parameters was based on a categorical numerical rating scale with an ordinal ranking, where zero represented optimal welfare and the scores increased as welfare levels declined (Hansen 2003; Barnard *et al.* 2016). Either binary or three- or four-point scales were used according to the distribution of measures (Appendix 2; Supplementary material). Each parameter was assigned a score which was subsequently aggregated to yield an overall score. All parameters were considered to have equal importance, with no weighting applied in the scoring process. The levels in scoring systems for parameters, such as break in asepsis and surgical and post-operative complications, were based on clinical criteria and definitions from peer-reviewed articles and textbooks (Airikkala-Otter *et al.* 2018; Bohling 2020; Digangi 2020).

Prior to the initiation of the main data collection, a draft list of welfare assessment parameters and associated scores was piloted by AS (Figure 1; stage 2) involving 26 dogs undergoing CVNR procedures over a five-day period. The aim was to assess the following: feasibility of data collection in a busy CNVR facility; relevance of the indicators chosen for this facility specifically; and the effectiveness of the scoring protocol in capturing each parameter range. During busy periods, it became clear it was not possible for AS to assess all parameters from all dogs as they moved through various CNVR steps. Therefore, the sample size was increased to 80 dogs to ensure complete data for at least 50% of the total data entries. All data entries during the pre- and post-training phase, including the staff training, were performed by AS.

### Delivery of the dog welfare assessment (stages 3 and 9)

An assessment of the individual welfare status of two separate groups of dogs was made as they moved through the CNVR process from catching to release. The first group was assessed before a staff training intervention (Figure 1; stage 3) and the second group after this training intervention (Figure 1; stage 9). FRDs undergoing routine CNVR were enrolled onto the study using a randomised approach on days 1 and 4 of each week, where every second dog was selected for the study. Each dog was assigned a random token number at the time of catching and a serial number at the time of data entry; these numbers were used to trace each animal through each step of the CNVR process. The assessment of parameters occurred at various time-points over several days as the dogs moved through each step. Once completed, both the welfare scores for each parameter and the combined, total scores for dogs assessed before and after the staff training intervention, were then compared.

### Staff training intervention (stages 4 to 8)

#### Analysis

After completion of the first dog welfare assessment, conducted in stage 3 (prior to staff training), the data were analysed and parameters scoring 15 or above were identified. This cut-off was considered to indicate sub-optimal welfare by the authors, thereby indicating where improvements were needed (Figure 1; stage 4). These were then used to inform the design of a targeted staff training intervention. Prior to its delivery, staff completed a paper-based assessment (Figure 1; stage 5) to evaluate their current knowledge and attitudes to be covered in the training intervention (Appendix 3; Supplementary material). A total of 14 questions were included: six regarding catching, transportation, and release; six concerning surgery and pre- and post-operative steps; and two regarding kennelling. The training intervention was then conducted by AS which comprised two, 1-h, theory-based lectures and four interactive sessions, including the practicing of correct procedures and group discussions (Figure 1; stage 6). Staff then completed an identical paper-based assessment (Figure 1; stage 7) and these scores were used to evaluate changes in their immediate knowledge and attitudes to the learning materials. For a period of three weeks after training, periodic, random spot-checks were performed on the staff by AS to check competency in the material included in the training, and to offer corrective feedback where required (Figure 1; stage 8).

#### Data capture

Data on the dog welfare indicators were recorded using a mobile device app designed by the WVS charity for the purpose of offline data collection in field conditions (Gibson *et al.* 2015). In addition to the welfare assessment parameters, the following data were also recorded: dogs’ age; sex; number of dogs caught and transported per batch; and operating surgeons’ experience: the latter refers to whether the surgery was conducted by a veterinary participant (less experienced) directly supervised by a WVS staff veterinarian, or by the actual WVS staff veterinarian (more experienced). Dogs less than three months of age were considered puppies, three to 12 months as juveniles and above one year as adults (Fielding *et al.* 2021). When logistically feasible, data were inputed into the app at the time of assessment; alternatively, paper records were compiled and transcribed to the app at a later stage. Data were synchronised to a cloud-based server for review and analysis via a secure website log-in.

#### Statistical analysis

Microsoft® Excel (2010, v 14.0.7173.5000) was used to store and manipulate the digital data securely. Minitab® (v 20.4.0.0) was used to generate graphs and perform statistical hypothesis testing. Normality was assessed using an Anderson-Darling normality test (Minitab®), whereby a *P*-value of < 0.05 indicated that the data were normally distributed. Generalised Linear Mixed Models (GLMM) were used to assess the effect of training on the scores for the 22 dog welfare parameters. As the data for the individual parameters were categorical, a multinomial logistic regression analysis test was run for each parameter using a logit-link function. The fixed effects were ‘assessment phase’ (pre- or post-training), sex of dog (female/male) and age of dog (adult/juvenile). For the parameters that are likely to be affected by the experience of the surgeon (i.e. P13, P14, P15 and P19), ‘surgeon experience’ (veterinary participant vs WVS staff veterinarian) was included as an additional fixed effect. The batch number (i.e. the ID given to the group of animals collected from one location on one day) was used as a random effect. The additional parameter of ‘total score’ was calculated for each animal; this was the summed value across all individual parameter scores but was only calculated for animals that had scores for each of the individual parameters (i.e. no missing values). The data were normally distributed, so this ‘total score’ parameter was analysed using a linear mixed model, with assessment phase, dog sex and dog age as fixed effects and batch as the random effect as previously. As repeated tests were carried out on the same dataset, a Bonferroni correction was applied. This meant that the threshold for significance was *P* = 0.003. These analyses were run in Genstat (v19; VSNi, Hemel Hempstead, UK).

## Results

A total of eighty-two dogs were assessed prior to the staff training intervention over a period of 13 days and eighty-one after the staff-training intervention over eleven days. Table 1 summarises the number of dogs assessed in each sex and age class. Note that the sample of dogs assessed in the pre-training stage (Figure 1; stage 3) was different from that sampled in the post-training stage (Figure 1; stage 9). As there was only one puppy in the pre-training stage, the data from this animal were removed from the dataset prior to further analysis. One dog with surgical complications and two dogs with transmissible venereal tumours, were diagnosed during the pre-training period; whilst they were included in the study, they were admitted for treatment and were subsequently released at a later stage. The total time taken to complete the assessment for one dog varied between 75 and 220 min; this was due to the variation in the time taken for each surgeon to complete the surgical procedure.

**Table 1.** Number, age and sex of two groups of free-roaming dogs included in a study to assess their welfare outcomes as they undergo CNVR before and after a staff training intervention in a training facility in India

	Pre-training			Post-training		
Age group	Male	Female	All	Male	Female	All
Adult	40	30	70	38	39	77
Juvenile	4	7	11	1	3	4
Total	44	37	81	39	42	81

### Welfare assessment scores

**Figure 3.**
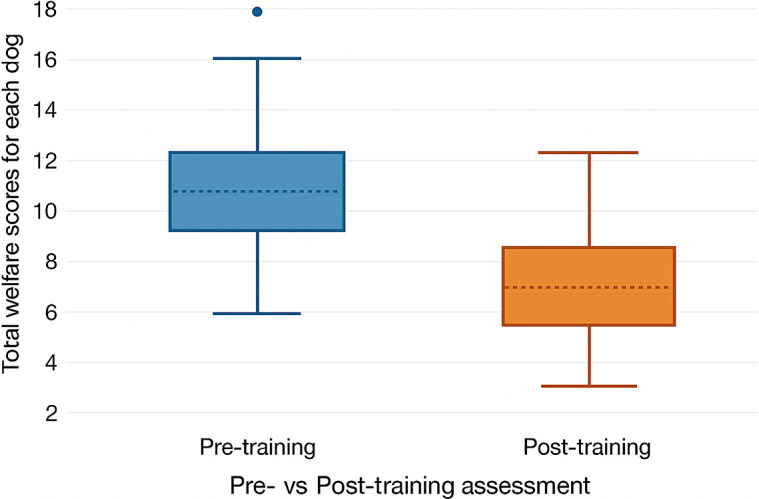
Boxplot to show the total scores for free-roaming dogs undergoing CNVR at a training facility in India, both before and after a staff training intervention. Blue denotes pre- and orange denotes post-training assessment scores.

Only animals with data for every parameter were included in the assessment of total scores, resulting in a total of 57 dogs included in the pre-training period and 56 dogs in the post-training period. Incomplete data were recorded for 24 dogs in the pre-training assessment and 25 dogs in the post-training assessment, with the number of missing parameters per dog ranging from 1 to 16. The total possible range for the total scores per dog was 0 to 49, with individual animal scores ranging from 2 to 18 in this study. Results of the statistical analyses of the individual parameters are summarised in Table 2. The total scores for the pre-training period were 607 (median 10), whilst the post-training score was 351 (median 6) (Figure 3). The total welfare scores were significantly lower after staff training intervention than before training (Wald = 74.15, ndf, ddf = 1, 13.2; *P* < 0.001 means [± SEM] [and range in brackets] for pre- and post-training = 10.6 [± 0.4] [5–18] and 6.3 [± 0.3] [2–12], respectively).

**Table 2.** Median scores, inter-quartile ranges (Q1–Q3), number of non-missing (N) and missing (N*) values for the individual parameter scores, and test statistics for the total welfare scores, of two groups of free-roaming dogs undergoing CNVR either before or after a staff training intervention, at a training facility in India. *P*-values in bold are significant after the Bonferroni correction was applied. Medians and IQRs are shown for the individual parameters as they were categorical variables, while the total scores could be considered as a continuous trait (*DNC – analysis did not converge)

Steps	Parameters		Pre-training period			Post-training period			Test Statistics	
			N (N*)	Q1-Q3	Median	N (N*)	Q1-Q3	Median	W value	*P*-value
CATCHING & TRANSPORT [Day 1]	P1	Space to stand and lie comfortably in transport	81 (1)	1–2	1	71 (10)	0–1	0	21.11	**<0.001**
	P2	Presence of injury due to catching/transport	82 (0)	0–2	0	81 (0)	0–0	0	1.98	0.161
	P3	Presence of lameness due to catching/transport	82 (0)	0–0	0	81 (0)	0–0	0	–	DNC
	P4	Blood on equipment	82 (0)	0–0	0	81 (0)	0–0	0	–	DNC
CAGE/HOLDING [Day 1 – 2]	P5	Physical injury in kennel	82 (0)	0–0	0	81 (0)	0–0	0	–	DNC
	P6	Pre-operative drinking water	81 (1)	0–1	0	81 (0)	0–0	0	13.13	**<0.001**
	P7	Vocalisation during sedation	70 (12)	0–1	0	78 (3)	0–1	0	1.57	0.230
	P8	Handler tests dog	70 (12)	0–1	1	78 (3)	0–1	0	18.20	**<0.001**
PRE -OPERATIVE [Day 1 – 2]	P9	Pre-operative examination	81 (1)	0–1	0	81 (0)	0–0	0	0.85	0.374
	P10	Ear notch bleeding	80 (2)	0–1	0	81 (0)	0–0	0	0.76	0.384
	P11	Measures to maintain body temperature during surgery	76 (6)	2–2	2	81 (0)	0–1	1	121.62	**<0.001**
SURGERY [Day 1 – 2]	P12	Vocalisation during surgery	75 (7)	0–0	0	81 (0)	0–0	0	0.29	0.588
	P13	Break in aseptic technique	73 (9)	0–0	0	79 (2)	0–0	0	1.92	0.185
	P14	Surgical complications	76 (6)	0–0	0	81 (0)	0–0	0	–	DNC
	P15	Surgery time	80 (2)	1–2.75	2	81 (0)	1–2	2	0.59	0.455
POST-OPERATIVE [Day 2 – 3]	P16	Post-operative temperature	80 (2)	1–2	1	80 (1)	1–1	1	10.58	0.005
	P17	Recovery time	80 (2)	0–0.75	0	81 (0)	0–2	1	15.35	**<0.001**
	P18	Post-operative drinking water	80 (2)	0–1	0	81 (0)	0–0	0	19.11	**<0.001**
RELEASE [Day 5 – 7]	P19	Post-operative complication	75 (7)	0–0	0	81 (0)	0–0	0	3.32	0.085
	P20	Wounds during transport for release	78 (4)	0–0	0	71 (10)	0–0	0	1.09	0.299
	P21	Lameness during transport for release	78 (4)	0–0	0	71 (10)	0–0	0	–	DNC
	P22	Released in a different location	78 (4)	0–0	0	71 (10)	0–0	0	1.64	0.203

Regarding the individual parameters, a significant reduction in scores (thereby indicating an improvement in welfare), was observed for the following parameters: the ‘space during transport’, ‘provision of pre-operative water’, ‘handler testing’, ‘measures to maintain temperature’, ‘recovery time’, ‘provision of post-operative water’ (*P* < 0.01). There was a statistical tendency for ‘post-operative temperature’ to improve between the pre- and post-training assessments. Female dogs had significantly higher (poorer) scores for ‘vocalisation during surgery’, ‘surgery time’ and ‘post-operative temperature’, while male dogs had higher scores for ‘measures to maintain temperature’ and ‘recovery time’ (*P* < 0.003). Female dogs also had higher total scores (Wald = 13.39, ddf, ddf = 1, 107.8; *P* < 0.001; means [± SEM]: females = 9.1 [± 0.5]; males = 7.8 [± 0.3]). There were no significant effects of age of dog on any single measure, or the total score (*P*-values all greater than Bonferroni adjusted value of *P* < 0.003).

The effect of surgeon’s experience (WVS staff veterinarian vs veterinary participant) was assessed for the relevant parameters assessing the surgery. The WVS staff veterinarians had lower scores than the veterinary participants for surgery time, indicating that the surgery took less time when performed by a staff veterinarian (Wald = 84.16, ndf, ddf = 1,109; *P* < 0.001). For the remaining parameters, there was no significant effect of surgeon experience.

### Staff training intervention and assessment

The steps in the CNVR process identified as requiring improvements involved several veterinary- and non-veterinary-related staff roles, although did not include veterinarians themselves. Subsequently, a total of 12 staff members were trained, comprising four catchers, two veterinary assistants cum catchers, five veterinary assistants and one driver. It should be noted that the staff veterinarians routinely receive ongoing training through the organisation as part of their Continuing Professional Development, and the training provided during the study itself did not pertain to their roles. The pre- and post-assessments and training were delivered to the staff according to their involvement in the procedures. Out of the 14 questions used in the assessment, six regarding catching, transportation, and release were answered by four catchers and one driver; six concerning surgery and pre- and post-operative steps were answered by five veterinary assistants; and two regarding kennelling were answered by all participants. The two staff members who served as both catchers and veterinary assistants responded to all 14 questions. Staff details (role and experience) and their assessment scores before and after the training intervention are shown in Table 3. After completion of the training intervention, a significant improvement was observed in the evaluation scores of staff compared to pre-training scores (*P* = 0.003). There was no correlation between the grades and experience (Spearman correlation: *r* = –0.100; *P* = 0.756).

**Table 3.** Job roles, experience and percentage assessment scores of staff members working in a training facility in India, both before and after a staff-training intervention. This forms part of a study to assess the impact of this training intervention on the welfare experience of free-roaming dogs undergoing the CNVR process

Staff member	Role	Experience	Pre-training score (%)	Post-training score (%)
1	Driver	3 months	3/8 (37.5%)	5/8 (62.5%)
2	Catcher	2 months	4/8 (50.0%)	4/8 (50.0%)
3	Catcher	1 year	3/8 (37.5%)	6/8 (75.0%)
4	Catcher	5 years	3/8 (37.5%)	4/8 (50.0%)
5	Catcher	6 years	2/8 (25.0%)	6/8 (75.0%)
6	Vet Assistant/Catcher	6 months	6/14 (42.9%)	10/14 (71.4%)
7	Vet Assistant/Catcher	2 years	6/14 (42.9%)	9/14 (64.3%)
8	Vet Assistant	4 years	6/8 (75.0 %)	8/8 (100.0%)
9	Vet Assistant	8 years	5/8(62.5%)	7/8 (87.5%)
10	Vet Assistant	14 years	5/8 (62.5%)	6/8 (75.0%)
11	Vet Assistant	16 years	4/8 (50.0%)	6/8 (75.0%)
12	Vet Assistant	17 years	6/8 (75.0%)	7/8 (87.5%)

## Discussion

This study describes the implementation of a comprehensive assessment protocol comprising parameters that evaluate the welfare status of FRDs at key steps in the CNVR process in a training centre in Goa, India. In addition, it demonstrates that the outcome can inform the compilation and delivery of a targeted staff training intervention in areas which, in turn, leads to an improvement in the overall welfare experience of individual dogs.

### Dog welfare assessment

Since welfare is the outcome of multifactorial effects, multiple variables were considered when formulating the study protocol encompassing the entire process from catching to release. Parameters were chosen from all key steps in the CNVR process (catching and transportation, cage/holding area, pre-operative period, surgery, post-operative period, and release) to ensure a comprehensive approach.

During CNVR, dogs are kennelled for short durations in unfamiliar surroundings with unfamiliar conspecifics; this causes an acute stress response (Bacon *et al.* 2017). Such stress can contribute to behavioural problems, such as anxiety, stereotypic behaviours, and stress-related aggression (Barnard *et al.* 2016; Hiby & Hiby 2017). Incorporating these behavioural indicators into a welfare audit, especially for FRDs, demands expertise among the staff to accurately interpret the behavioural indicators which is challenging (Bacon *et al.* 2019b). Several ABMs and RBMs used in the Shelter Quality Protocol (Arena *et al.* 2018) and by Bacon *et al.* (2019a) to assess these behaviours, were excluded from this study as they were considered non-discriminatory in this setting, and potentially difficult to assess reliably in a ‘spot-check’ scenario. Negative behavioural parameters, such as fear, escape behaviour, and inter-dog aggression, were also excluded; this was because the majority of the FRDs were expected to show these behaviours routinely as their natural response to human handling (Reece *et al.* 2012; Raudies *et al.* 2021); and therefore, considered unlikely to be an accurate indication of poor welfare during the CNVR process.

The study did incorporate several behavioural parameters. Excessive vocalisation during handling can have a detrimental effect on the individual as well as other dogs housed in the kennel (Kiddie & Collins 2014; Arena *et al.* 2018; Berteselli *et al.* 2019) and was included as an ABM. Parameters such as ‘animal handler test’ and ‘vocalisation during sedation’ measured the approach behaviour of a dog towards a staff member and were used to assess the positive and negative emotional responses of the animal (Arhant & Troxler 2014; Bacon *et al.* 2019a; Menchetti *et al.* 2019). Arhant and Troxler (2014) considered a test assessing the behaviour of animals towards humans as a valid measure of the quality of the human-animal relationship. At this facility, efforts are taken to mitigate stressors associated with confinement by keeping the post-surgery hospitalisation period to a minimum (Airikkala-Otter *et al.* 2018).

Several other parameters were excluded from the study. These included longer-term health measures outwith the CNVR time-frame (Barnard *et al.* 2014), such as changes in body condition score, coat condition or signs of infection, RBMs, such as presence of an emergency crash kit, hygiene control and multimodal analgesia, which were already routinely incorporated at the facility and post-operative assessments of wound healing and pain levels which were already routinely assessed (Airikkala-Otter *et al.* 2018). However, these parameters may be appropriate for other CNVR settings.

A dedicated anaesthetist is one of the MBMs that is critical in reducing mortalities in the CNVR process (Looney *et al.* 2008; Bacon *et al.* 2019b); however, this was not included in this study due to staffing constraints. Since a light plane of anaesthesia was also an indication of negative welfare (Bacon *et al.* 2017), the parameter ‘dedicated anaesthetist’ was replaced by the parameter ‘vocalisation during surgery’ to ensure the animals’ welfare was not compromised during the procedure by indirectly measuring the depth of anaesthesia and level of analgesia. However, the WVS surgery team ensured that the animals were monitored by a ‘shared’ trained veterinary theatre assistant, which is one of the factors contributing to the low mortality rate at the centre (Airikkala-Otter *et al.* 2018). Parameters such as break in asepsis, surgical complications, and surgery time evaluated intra-operative welfare.

Maintenance of body temperature is essential for upholding welfare (Berteselli *et al.* 2019). Shivering/huddling/panting indicate abnormal body temperature and the use of bedding material has been used in Shelter Quality Protocol as a measure of the good housing welfare criterion (Arena *et al.* 2018; Berteselli *et al.* 2019). These indicators were not considered in this study as the average, environmental temperature in Goa is 27°C with 75.8% humidity (Fielding *et al.* 2021); therefore, the occurrence of huddling/panting is minimal. Moreover, bedding material was restricted to hypothermic animals, to reduce the chances of nosocomial infections through fomites among healthy individuals (Digangi 2020). Instead, maintenance of body temperature was assessed using post-operative temperature score and recovery time.

Incidents such as lameness during catching and release, injuries in the kennel and the presence of blood on the equipment, were observed rarely during the study; this was predicted by AS from his experience with the CNVR process at the facility. However, these animal-based parameters were included as their occurrence signifies serious welfare concerns (Barnard *et al.* 2014; Grandin 2015) and require further research; a larger sample size may be required to explore more extensively their sensitivity to catching and handling. Parameter space to stand/lie comfortably during transport was assessed to check for overcrowding during transport.

Since the study was conducted at a training centre, it was well equipped with resources such as an inhalant anaesthesia machine, multiple isolation wards and holding kennels, dedicated operating theatre, adequate number of experienced veterinary assistants and surgeons, and basic diagnostic machines that contributed to high levels of patient care; these were, therefore, excluded. Selecting sufficient parameters involving all steps of the process that are contextually relevant to each facility, whilst ensuring it remains feasible and practical to complete, will result in a comprehensive evaluation of the welfare status of the animal.

### Effect of staff training intervention on welfare parameters

The staff training intervention significantly improved several parameters in the following key areas: space to stand and lie comfortably in transport (catching and transportation); availability of water pre-operatively and the handler test (pre-operative period); measures to maintain body temperature during surgery (surgery); and post-operative temperature and post-operative drinking water (post-operative period).

Whilst the staff training intervention addressed the requirement for improved space allowance within the transporting vehicle, the significant improvement observed post-training may have been influenced by factors outwith the intervention itself. Due to the COVID-19 pandemic, the number of dogs caught and, therefore, the number of surgeries performed daily, was reduced in the post-training compared to the pre-training phase. Nevertheless, this highlights the importance of providing adequate space for unfamiliar dogs in CNVR facilities, thereby reducing the likelihood of direct conflicts and concomitant increased stress and injury. There are several potentially deleterious effects of increased stress, both on behaviour and physiology; one such example is an increase in an animals’ requirements for water intake (Velarde & Dalmau 2012). The importance of providing sufficient drinking water at the appropriate times both pre- and post-operatively was emphasised during the staff training intervention and significant improvements in welfare outcomes were observed as a result. Other factors can also increase water intake requirements, such as environmental temperature during transport (Velarde & Dalmau 2012), and the use of anaesthetic drugs during surgery (Grubb *et al.* 2020). Barnard *et al.* (2016) found that inadequate drinking water led to a three-fold increase in the prevalence of diarrhoea in dogs. However, facilities must be mindful that, even when water is provided, dogs may be reluctant to drink due to factors such as unfamiliar surroundings or human interaction. This emphasises the need for a holistic approach to individual dog welfare assessment through considering multiple parameters and how these may impact each other.

Unsurprisingly, the manner in which staff handled and interacted with the dogs affected the animals’ welfare scores, and significant improvements in welfare were demonstrated after the training intervention addressed such techniques. An individual dog’s behaviour when encountering people is likely based on factors such as personality and previous experiences with humans; it is known that FRDs can exhibit negative behavioural responses towards human contact (Gibson *et al.* 2015). It can be argued therefore that each animal should be provided sufficient time to acclimate to people prior to being approached. However, counter-arguments exist for minimising contact time (Muri *et al.* 2013) and quickly sedating the animal. Both approaches were demonstrated to veterinary assistants during practical sessions, with the advice to minimise contact time for aggressive dogs (Barnard *et al.* 2016) and provide adequate acclimatisation time for timid and juvenile dogs before sedation (Arhant & Troxler 2014). Adequate staff training is key to ensuring they can correctly recognise different dog behaviours and act accordingly.

The data collection process was carried out by a single observer which, for logistical reasons, sometimes precluded the concurrent assessment of individual dogs at different steps in the CNVR process. This typically occurred when the centre was very busy; as a result, incomplete datasets were generated for some of the dogs. Given that the overall welfare scores were calculated by aggregating values across multiple parameters, the presence of missing data rendered the total scores not comparable with complete datasets. Therefore, these cases were excluded from the final dataset and statistical analysis. However, as these omissions occurred randomly and were not related to the attributes of individual dogs, such as their behaviour, removal of these individuals from the final dataset was not considered to bias the results.

The parameter that showed the highest improvement in scores post-training was the ‘measures to maintain body temperature during surgery’. In Airikkala-Otter *et al*.’s (2018) study, hypothermia was identified as one of the spay neuter complications, affecting 0.7% of the dogs in the study sample. To reduce the incidence of hypothermia, this study utilised interventions such as using warm fluids, gloves with hot water over the infusion set, covering body extremities with socks or paper, and maintaining the surgical theatre temperature around 25°C (Redondo *et al.* 2012). These actions most likely contributed to the improvement in the post-operative temperature scores as well during the post-training assessment.

There was no significant improvement in assessment scores for surgery and post-operative complication parameters. This observation was consistent with those reported by Airikkala-Otter *et al.* (2018). This is likely due to the standardisation of the surgery protocols in accordance with current protocols and understanding of good welfare practices. Inexperienced surgeons, including veterinary students, have been associated with a higher incidence of break in asepsis, surgery complications, wound dehiscence, and post-operative complications, such as remnant ovary syndrome and haemoabdomen (Bohling 2020; Digangi 2020). Bacon *et al.* (2019a) viewed surgery carried out by inexperienced veterinarians or students as delivering a lower standard of welfare.

Surprisingly, the ‘recovery time’ increased after the staff training intervention, despite improved efforts to maintain body temperature. The reason for this is unclear; however, post-training data were collected in January, when the outdoor temperature was lower, which may have influenced the results. Seasonality has been shown to influence such parameters (Griffin *et al.* 2016; Grubb *et al.* 2020).

A significant difference in post-operative complication scores was observed between surgeries performed by veterinary participants and WVS staff veterinarians. In this study, veterinary participants were inexperienced and therefore took longer to complete the surgeries, which is to be expected at a training facility. There are studies that have found a similar correlation between longer surgery time and post-operative complication rate (Reece *et al.* 2012). This may result from increased trauma during tissue handling, excessive retraction, and tissue dehydration by inexperienced surgeons that resulted in greater susceptibility to infection (Vasseur *et al.* 1988). However, observations made by Airikkala-Otter *et al.* (2018) at a similar WVS training centre did not find a correlation between longer surgery time and surgical site complications.

Our findings demonstrated that improving staff knowledge through a targeted training intervention, combining both didactic teaching and practical sessions, resulted in an enhanced welfare experience for individual dogs during the CNVR process. Whilst this focused upon demonstrating short-term outcomes, it may be integrated into a facility’s protocols to ensure sustained standards of welfare through regular assessment and training. Hiby *et al.* (2017) reported that CNVR staff can have limited knowledge of welfare assessments and may be unaware of the most meaningful or cost-effective indicators to monitor in the long term. Effective staff training is therefore central to ensuring high welfare standards for dogs in CNVR programmes (Bacon *et al.* 2017); and a preventative ‘duty of care’ approach, combined with staff training, can mitigate short-term welfare issues (Bacon *et al.* 2019a).

The amount of experience held by individual staff was not associated with their grades; this is in accordance with the findings of Mornement *et al.* (2010) who showed that highly experienced staff were not confident in assessing animal welfare using the protocols practised in their shelters. This underscores the importance of regular training for all staff, regardless of their overall experience (Kagan *et al.* 2015). Other studies have shown that staff in a CNVR setting can find some aspects of welfare assessment challenging, such as identifying subtle behaviour changes during pain and stress, with possible causes including a lack of training and cognitive dissonance (Bacon *et al.* 2019a). Furthermore, the quality of life of animals in an institution is greatly influenced by the staff and their relationship with them (Kagan *et al.* 2015). Certainly, attitudes and behaviours are inter-related and a positive attitude towards animals from shelter staff has been shown to lead to better welfare for dogs (Arhant & Troxler 2014). Whilst these specific components were not included in the assessment of the staff during this study, they could form the basis for future investigations to assess their potential impact on post-assessment welfare scores.

Visual training methodologies were used to minimise the potential challenges during translation into the different languages used by the staff, as followed by Howard and Reed (2015) in their study. Pictures from different CNVR facilities that illustrated scenarios where welfare was compromised were used in the presentation. Follow-up training during the spot-check period used corrective feedback through verbal communication and demonstrations to rectify any mistakes. The training demonstrated proper net-tying techniques post-capture to ensure that the animal can move or stand inside the net but cannot walk.

To the authors’ knowledge, this is the first study to evaluate the effect of focused staff training on the individual welfare experience of free-roaming dogs undergoing CNVR in a training facility in India.

### Limitations of the study/future work

A number of limitations were identified in this study. Some parameters formed part of previously validated protocols (Barnard *et al.* 2016; Bacon *et al.* 2019a; Berteselli *et al.* 2021), but indicators such as space to stand and lie during transport, vocalisation during sedation, handler-tests dog, vocalisation during surgery, surgical complications, ear-notch bleeding, surgery time, recovery time, post-operative temperature, and post-operative complications were not validated before inclusion.

Whilst it was not feasible to compare welfare scores from the same sample of dogs both before and after the training intervention, the results nevertheless showed improved welfare outcomes for dogs undergoing CNVR at the facility; such parameters included greater space during transport and availability of pre-operative drinking water, which do not necessitate direct reassessment of the same individuals to demonstrate improvements. In both the pre- and post-training welfare assessment, welfare outcomes from two distinct groups were used to compare the effectiveness of the staff training intervention.

The post-training evaluation of the staff was undertaken immediately after the training, which limited the study to assessment of short-term retention of knowledge. Re-assessing the impact of training by conducting post-training evaluations at a later date would have estimated the accurate level of long-term knowledge retention, and potentially a change in working practices, the latter demonstrating the application of retained knowledge in their daily work. However, these evaluations were out of the scope of this study. Long-term follow-up is required to validate the training programme, and to understand if the knowledge attained will affect sustained behavioural changes.

WVS staff were not directly involved in the study other than undertaking their routine clinical duties. However, their awareness of it may have biased their decision-making and practices, especially after the training. In addition, as AS was the only assessor for the study, his presence could have further influenced the staff to follow the guidelines mentioned during the training, thereby affecting the scores.

Potential bias could have been avoided by engaging an independent assessor who was blind to the application of the training period. Furthermore, the inclusion of a control group of untrained staff to compare against the trained group when assessing the educational intervention would have improved the methodology. Lastly, feedback about the training was not collected from the staff. Arhant and Troxler (2014) found via a staff questionnaire that a positive attitude towards animals from the staff ensured better welfare for the dogs. Hence, a similar feedback questionnaire post-training could have helped in improving the future training (Steneroden *et al.* 2011) and would also have served as an indicator of their general attitude to animal welfare (Muri *et al.* 2013).

Whilst the protocol used in this study had its limitations, it combined measures to identify welfare issues in the CNVR process and was performed at minimal cost by a single assessor in three months. The resulting score was easy to interpret for both technical and non-technical personnel, making it relevant to the wider dog population management community.

### Animal welfare implications

In summary, this study demonstrates that even in well-run CNVR programmes, it is possible to identify parameters that can improve the overall welfare status of a free-roaming dog going through the CNVR process. These can include a range of welfare indicators comprising animal, resource, and management factors that have been identified as relevant to the individual setting, and that are practical and simple to implement. The findings can then be used to inform and conduct a targeted, staff training intervention, the outcome of which subsequently improved the welfare experience of the dogs. To the authors’ knowledge, this is the first time a study has been conducted assessing the impact of staff training on CNVR animal welfare. Furthermore, it shows that evidence-informed staff training can be undertaken and effectively implemented in busy environments, such as a high-volume spay-neuter facility.

These findings can be used by other CNVR facilities to incorporate both dog welfare assessments and staff training as part of the standard operating procedures to ensure patient care and welfare is upheld long term, enabling facilities to provide evidence of active and ongoing engagement of quality standards for the welfare of animals under their care.

## Supporting information

Susheelan et al. supplementary material Susheelan et al. supplementary material

## Supplementary material

The supplementary material for this article can be found at http://doi.org/10.1017/awf.2025.10049.
