# Supplementary material for: The effect of staff training on welfare outcomes of free-roaming dogs in a catch-neuter-vaccinate-release facility in India
Source: Anim Welf. 2025 Nov 13;34:e74. doi: 10.1017/awf.2025.10049 (PMC12645508; doi:10.1017/awf.2025.10049)
Supplement: Susheelan et al. supplementary material [file S0962728625100493sup001.pdf]

The effect of staff training on welfare outcomes of free-roaming dogs in a catch-neuter-vaccinate-release facility in India: Supplementary material

Aswin Susheelanhttps://orcid.org/0009-0001-4464-1200 [1], Emma L Raynerhttps://orcid.org/0009-0009-6314-3483 [2], Luke Gamble [2], Marie J Haskell https://orcid.org/0000-0001-9373-0624 [3]

[1] Worldwide Veterinary Service, Mission Rabies, Mumbai, India, 400703

[2] Worldwide Veterinary Service, 4, Castle Street, Cranborne, Dorset BH21 5PZ, UK

[3] SRUC (Scotland's Rural College), West Mains Road, Edinburgh EH9 3JG, UK

Author for correspondence: Marie J Haskell, email: marie.haskell@sruc.ac.uk

Appendix 1

Initial set of parameters that were identified to assess the welfare of free-roaming dogs undergoing CNVR at a training centre in India. The 22 parameters included in the study are indicated in bold.

CNVR Programme

1. Facilities for treating additional medical conditions (Bacon et al 2019)
2. Crash kit and drugs available (Bacon et al 2019)
3. Biosecurity/hygiene protocol in place (Bacon et al 2019)
4. Positive community feedback on dogs (Bacon et al 2019)
5. Regular/continued training for CNVR staff (Bohling 2020, Grubb et al 2020, Personal)

Capture and Transport

6. Body Condition Score/ signs of disease (Barnard et al 2016, Berteselli et al 2021)
7. **Presence of injury due to capture/transport** (Barnard et al 2016)
8. **Presence of lameness due to capture/transport** (Berteselli et al 2021)
9. Mortality at capture/transport (Barnard et al 2016, Bacon et al 2019)
10. Use of capture equipment (Bacon et al 2019)
11. **Blood on equipment** (Bacon et al 2019)
12. **Space to stand and lie comfortably in transport** (Barnard et al 2016, Bacon et al 2019)

13. Fear/aggression to catcher/handler (Bacon et al 2019)

Cage/holding area indicators

14. **Physical injury** (Barnard et al 2016, Bacon et al 2019)
15. Kennel space (Barnard et al 2016)
16. **Drinking water in kennels** (Barnard et al 2016, Bacon et al 2019)
17. Bedding material (Barnard et al 2016)
18. Signs of thermal discomfort (Barnard et al 2016, Berteselli et al 2021)
19. **Vocalisation** (Bacon et al 2019)

Pre-operative

20. **Handler tests dog** (Arhant and Troxler 2014, Bacon et al 2019)
21. Fear/aggression to handler (Barnard et al 2016, Grubb et al 2020, Berteselli et al 2021)
22. **Pre-anaesthetic examination** (Airikkala-Otter et al 2018, Grubb et al 2020)
23. Analgesic drug administration (Bacon et al 2019, Grubb et al 2020)
24. Surgical checklist (Bohling 2020, Digangi 2020)

Surgery

25. **Vocalisation during surgery** (Bacon et al 2019)
26. Movement of head or forelimbs during surgery (Bacon et al 2019)
27. **Break in aseptic technique** (Airikkala-Otter et al 2018, Digangi 2020)
28. Surgery performed by student or inexperienced veterinarian (Bacon et al 2019, Bohling 2020)
29. Dedicated anaesthetic monitoring person (Airikkala-Otter et al 2018, Bacon et al 2019)
30. **Excessive surgical time** (Reece et al 2012, Bacon et al 2019)
31. **Surgical complications** (Vasseur et al 1988, Airikkala-Otter et al 2018, Bohling 2020)
32. **Ear tag placed/bleeding** (Bacon et al 2019, Personal)
33. **Hypothermia during surgery** (Redondo et al 2012, Personal)

Post-operative period

34. **Drinking water in kennels** (Barnard et al 2016, Bacon et al 2019)
35. Post-operative analgesia (Airikkala-Otter et al 2018, Bacon et al 2019)
36. **Post-operative hypothermia** (Redondo et al 2012)
37. Individual post-operative assessment (Bohling 2020)
38. Bedding material (Barnard et al 2016)
39. **Poor quality recovery** (Bacon et al 2019)
40. Pain behaviour (Bacon et al 2019)

41. Mortality & post-mortem examination (Vasseur et al 1988, Bohling 2020)

Release

42. Released prior to gaining full motor control or alertness (Bacon et al 2019)

43. Individual assessment prior to release (Bacon et al 2019)

44. Reduced activity/physical impairment (Bacon et al 2019)

45. **Presence of post-operative complication/s** (Bacon et al 2019, Bohling 2020)

46. Released prior to food/water offered (Barnard et al 2016, Bacon et al 2019)

47. Blood on equipment (Bacon et al 2019)

48. **Presence of injury due to transport** (Barnard et al 2016, Bacon et al 2019)

49. **Presence of lameness due to transport** (Berteselli et al 2021)

50. **Released in a different location** (Bacon et al 2019)

References

- Airikkala-Otter, I., Gamble, L., Mazeri, S., Handel, I.G., Bronsvoort, B.D.C., Mellanby, R.J. and Meunier, N.V., 2018. Investigation of short-term surgical complications in a low-resource, high-volume dog sterilisation clinic in India. *BMC veterinary research, 14(1)*: 1-8.

- Arhant C and Troxler J 2014 Approach behaviour of shelter dogs and its relationships with the attitudes of shelter staff to dogs. *Applied Animal Behaviour Science 160*: 116–126.

- Bacon, H., Walters, H., Vancia, V., Connelly, L. and Waran, N., 2019. Development of a robust canine welfare assessment protocol for use in dog (Canis familiaris) catch-neuter-return (CNR) programmes. *Animals, 9(8)*: 564.

- Barnard S, Pedernera C, Candeloro L, Ferri N, Velarde A and Villa PD 2016 Development of a new welfare assessment protocol for practical application in long-term dog shelters. *Veterinary Record, 178(1)*: 18. https://doi.org/10.1136/vr.103336

- Berteselli GV, Rapagnà C, Salini R, Badagliacca P, Bellucci F, Iannino F and Villa PD 2021 A Pilot Study to Develop an Assessment Tool for Dogs Undergoing Trap-Neuter-Release (TNR) in Italy. An Overview on the National Implementation of TNR Programmes. *Animals, 11(11)*: 3107.

- Bohling MW 2020 Complications in Spay and Neuter Surgery Complications of Ovariohysterectomy: *Veterinary Clinics: Small Animal Practice, 41(5)*: 1023-1039

- Digangi BA 2020 Is Asepsis Really a Requirement for Spay–Neuter Surgery? High-Quality, High-Volume Spay and Neuter and Other Shelter Surgeries: 65-88

- Grubb T, Sager J, Analgesia VTSA, Gaynor JS, Montgomery E, Parker JA, Shafford H and Tearney C 2020 *AAHA Anesthesia and Monitoring Guidelines for Dogs and Cats*: 59–82.

- Redondo JI, Suesta P, Serra I, Soler C, Soler G and Gil L 2012 Retrospective study of the prevalence of postanaesthetic hypothermia in dogs. Veterinary Record, 171(15): 374-374

- Reece JF, Nimesh MK, Wyllie RE, Jones AK and Dennison AW 2012 Papers Description and evaluation of a right flank, mini-laparotomy approach to canine ovariohysterectomy. *Veterinary Record, 171(10)*: 248-248

- Vasseur, P.B., Levy, J., Dowd, E. And Eliot, J., 1988. Surgical wound infection rates in dogs and cats data from a teaching hospital. *Veterinary surgery, 17(2)*: 60-64.

Appendix 2- Dog welfare assessment form with parameters and scoring system used to assess the welfare of free-roaming dogs undergoing CNVR at a training centre in India.

a. Animal ID
b. Token number
c. Sex – 1. Male 2. Female
d. Age – 1. Adult 2. Juvenile 3. Puppy
e. Total number of dogs caught

1. Space to stand and lie comfortably in transport (P1)

0 no physical contact with other dogs
1 physical contact with one dog
2 physical contacts with 2 dogs
3 physical contacts with more than 2 dogs

2. Presence of injury due to catching/transport (P2)

0 absent
1 wound involving skin
2 wound involving skin and subcutaneous
3 wound involving muscle and bones

3. Presence of lameness due to catching/transport (P3)

0 absent
1 limping – weight bearing
2 limping – non weight bearing
3 recumbent

4. Blood on equipment (P4)

0 absent
1 presence of blood

5. Physical injury in kennel (P5)

0 absent
1 wound involving skin
2 wound involving skin and sub-cutaneous
3 wound involving muscle and bones

6. Pre-operative drinking water (P6)

0 present, clean
1 present, dirty
2 absent

7. Vocalisation during sedation (P7)

0 no vocalisation
1 intermittent vocalisation
2 constant vocalisations

8. Handler tests dog (P8)

0 waits until the dog relaxes, and then sedate
1 sedate straight away

9. Pre-operative examination (P9)

0 the medicines are administered after proper examination of the animal
1 no physical examination of the dog

10. Ear notch bleeding (P10)

0 no bleeding
1 bleeding before recovery
2 bleeding after recovery

11. Measures to maintain body temperature during surgery (P11)

0 use of all 4 measures
1 use of 2 to 3 measures
2 use of less than two measures
(4 parameters - warm fluids, hot hands, extremities covered, air condition temperature kept at room temperature)

12. Vocalisation during surgery (P12)

0 no vocalisation during surgery
1 single vocalisation during surgery
2 two or more vocalisation events during surgery

f. Surgeon – 1. WVS vet 2. Participant

13. Break in aseptic technique (P13)

0 no break in asepsis – clean surgery
1 minor break – clean contaminated surgery
2 major break – contaminated and dirty surgery
Clean - gastrointestinal, or genitourinary tracts were not entered
Clean Contaminated - genitourinary tract, intestinal tract, or oropharyngeal cavity was entered, but without unusual contamination
Contaminated - operations with a major break in sterile technique, and incisions encountering acute, nonpurulent inflammation
Dirty - perforated viscera

14. Surgical complications (P14)

0 no complication
1 complication present
Complications are haemorrhage, nick to bladder, spleen, intestine, iatrogenic ureteral trauma

15. Surgery time (P15)

Castrate:

0 within 20 mins
1 over 21 and up to 40
2 over 41 and up to 60
3 over 61

Spay

0 within 30 mins
1 over 31 and up to 60
2 over 61 and up to 90
3 over 91

16. Post-operative temperature (P16)

0 – more than 100F
1- 99.9 to 98F
2- 97.9 to 96F
3 – less than 96F

17. Recovery time (P17)

0 within 20 mins
1 over 21 and up to 30 mins
2 over 31 and up to 40 mins
3 over 41 mins

18. Post-operative Drinking water (P18)

0 present, clean
1 present dirty
2 absent

19. Presence of post-operative complication before release (P19)

0 no complication
1 mild (Redness, itching, licking, or mild seroma present but no medication was required)
2 moderate (Surgical complications requiring pain medication and/or antibiotics)
3 severe (Anaesthesia required to repair surgical complications such as a dehiscence or hematoma)

20. Wounds during transport for release (P20)

0 absent
1 wound involving skin
2 wound involving skin and subcutaneous
3 wound involving muscle and bones

21. Lameness during transport for release (P21)

0 absent
1 limping – weight bearing
2 limping – non weight bearing
3 recumbent

22. Released in a different location (P22)

0 released at the exact spot
1 released at a different spot

Appendix 3 - Paper-based assessment questionnaire answered by the staff at a training centre in India to evaluate their knowledge and attitudes regarding animal welfare of dogs undergoing CNVR process.

Answered by Catchers and Catcher cum veterinary assistant

1. When the animal is inside the net, the net must be tied tight/loose/medium

A. tight – can't move
B. loose – can move and can walk around
C. medium – can stand but cannot move around
D. depends on the number of dogs in the vehicle
E. I don't know
Correct answer – C

2. When an animal is tied inside the net how can it cause injuries

A. by skin rubbing against the net when tied tightly
B. from the floor while vehicle is moving
C. when the animal tries to escape by chewing through the net
D. all the above
E. I don't know
Correct answer – D

3. What can be done to reduce the injuries while an animal is inside the net

A. don't tie the net tight
B. use a good quality net so that the animal cannot chew through the net easily and bruise themselves
C. transfer the dog to another net if there is a hole made in the net by the dog
D. all the above
E. I don't know
Correct answer – D

4. How many dogs can be transported without causing discomfort to dogs in our vehicle

A. 5-10
B. 11-15
C. 15+
D. As many as possible
E. Not sure
Correct answer – A

5. What are the signs of distress in an animal in kennel

A. barking, growling
B. howling continuously
C. moving away
D. chewing bars/wires of the kennel
E. all the above

Correct answer – E

6. Explain why it is important to release a neutered dog in the same location as it was caught.

A. gets attacked by other dogs, do not get food
B. it will not affect the animal, the dog will go back to his or her territory
C. not sure what happens after release
Correct answer - A

7. How far can an animal be released from the spot he was caught?

Correct answer – exactly the same spot

<u>Answered by Veterinary assistants</u>

8. Should clean drinking water be available to dogs while in kennel?

A. yes
B. no
C. depends on the climate and time of the day
D. depends on whether it is pre or post - surgery
E. don't know
Correct answer – A

9. How to approach an animal during sedation

A. don't waste time, sedate straight away
B. approach gently, let the animal relax, and then sedate
C. depends how busy the day is
D. depends on the temperament of the dog
E. don't know
Correct answer – B

10. Why is it important to perform a pre-operative exam prior to surgery?

A. makes sure the dog is healthy and fit for the surgery
B. it is not required as the dog is checked by the anaesthetist before surgery
C. depends on the staff availability
D. don't know
Correct answer – A

11. How often can an animal vocalise during surgery and considered normal

Correct answer – never

12. How often to give anaesthesia

A. depends on blink reflex, jaw tone
B. when an animal starts to move
C. when an animal makes sound

D. don’t know
Correct answer – A

13. What is the normal body temperature of a dog

Correct answer – 101–102-degree Fahrenheit

14 List the methods that can help maintain the patient’s body temperature during surgery

A. covering the feet and warm fluids
B. keeping room temperature cold
C. giving antibiotics before surgery
D. all the above
E. don’t know
Correct answer - D
